# Identifying candidate genes for variation in sleep-related quantitative traits

**DOI:** 10.1186/1471-2105-15-S10-P13

**Published:** 2014-09-29

**Authors:** Shreyas Joshi, Bruce O'Hara

**Affiliations:** 1 Department of Biology, University of Kentucky, Lexington, KY 40506, USA

## Background

Humans spend approximately one third of their lives sleeping, but compared with other biological processes, most of the molecular and genetic aspects of sleep have not been elucidated. A non-existent gene ontology and lack of a dedicated database containing a comprehensive list of sleep-related genes and their function presents a hurdle for sleep researchers.

## Materials and methods

Using a two-pronged approach to solve this problem, publicly available microarray data from NCBI GEO (National Center for Biotechnology Information – Gene Expression Omnibus) database was used to develop a list of sleep-related genes for traits of interest. The data were analyzed using R Bioconductor and custom Perl scripts. The genes from this list were then matched with the genes in QTL (Quantitative Trait Loci) for the trait. The genes within the QTL chromosomal region matching any in the list of sleep-related genes were considered as potential candidates for causing variations in the quantitative trait.

## Results

Here we present the results for our study conducted for sleep deprivation (SD) using this approach. 227 genes were identified which showed significant differential expression after 3, 6, 9 and 12 hours of sleep deprivation in three mouse strains. We were able to identify 4 candidate genes in Dps1 QTL, 2 in Dps2, and 9 genes in Dps3. Dps loci are the QTL associated with delta power in slow wave sleep [1]. The list also contains Homer1 which has already been established as a molecular correlate of sleep loss [2]. The advantage with this approach is that it provides more information and cross support than a simple list of sleep-related candidate genes. The association of information about genes with their function and role in sleep can help in forming sleep-specific gene ontologies, which would be useful for sleep researchers.
